# Thermodiffusion in multicomponent *n*-alkane mixtures

**DOI:** 10.1038/s41526-017-0026-8

**Published:** 2017-08-11

**Authors:** Guillaume Galliero, Henri Bataller, Jean-Patrick Bazile, Joseph Diaz, Fabrizio Croccolo, Hai Hoang, Romain Vermorel, Pierre-Arnaud Artola, Bernard Rousseau, Velisa Vesovic, M. Mounir Bou-Ali, José M. Ortiz de Zárate, Shenghua Xu, Ke Zhang, François Montel, Antonio Verga, Olivier Minster

**Affiliations:** 1 Laboratoire des Fluides Complexes et leurs Réservoirs-IPRA, E2S, UMR5150, Univ Pau & Pays Adour/CNRS/TOTAL, 64000 Pau, France; 2 Centre National d’Etudes Spatiales (CNES) 2, Place Maurice Quentin, 75001 Paris, France; 3 Laboratoire de Chimie-Physique, UMR 8000 CNRS, Université Paris-Sud, Orsay, France; 4 Department of Earth Science and Engineering, Imperial College London, London, UK; 5 MGEP Mondragon GoiEskola Politeknikoa, Mechanical and Industrial Manufacturing Department, Mondragon, Spain; 6 Departamento de Física Aplicada I. Universidad Complutense, Madrid, Spain; 7 Key Laboratory of Microgravity, Institute of Mechanics, Chinese Academy of Science, Beijing, China; 8 State Key Laboratory of Enhanced Oil Recovery (Research Institute of Petroleum Exploration & Development), CNPC, Beijing, China; 9 TOTAL Exploration Production, Pau, France; 10 European Space Agency, ESTEC, Noordwijk, The Netherlands

## Abstract

Compositional grading within a mixture has a strong impact on the evaluation of the pre-exploitation distribution of hydrocarbons in underground layers and sediments. Thermodiffusion, which leads to a partial diffusive separation of species in a mixture due to the geothermal gradient, is thought to play an important role in determining the distribution of species in a reservoir. However, despite recent progress, thermodiffusion is still difficult to measure and model in multicomponent mixtures. In this work, we report on experimental investigations of the thermodiffusion of multicomponent *n*-alkane mixtures at pressure above 30 MPa. The experiments have been conducted in space onboard the Shi Jian 10 spacecraft so as to isolate the studied phenomena from convection. For the two exploitable cells, containing a ternary liquid mixture and a condensate gas, measurements have shown that the lightest and heaviest species had a tendency to migrate, relatively to the rest of the species, to the hot and cold region, respectively. These trends have been confirmed by molecular dynamics simulations. The measured condensate gas data have been used to quantify the influence of thermodiffusion on the initial fluid distribution of an idealised one dimension reservoir. The results obtained indicate that thermodiffusion tends to noticeably counteract the influence of gravitational segregation on the vertical distribution of species, which could result in an unstable fluid column. This confirms that, in oil and gas reservoirs, the availability of thermodiffusion data for multicomponent mixtures is crucial for a correct evaluation of the initial state fluid distribution.

## Microgravity simulators: improving oil field assessments

To support oil and gas exploration, researchers sent hydrocarbon mixtures into space to obtain accurate data on how each component behaves. The group—led by Guillaume Galliero from the University of Pau and Pays de l’Adour, France—wanted to study the effect of temperature on the movement of individual hydrocarbons in mixtures under typical reservoir conditions. Eliminating the effects of gravity allowed them to collect more accurate data than has previously been obtained. The team showed that thermodiffusion has a large impact on the distribution of hydrocarbon reservoirs under the ground. They state that thermodiffusion should therefore be considered in computer models that assess analytical data collected at potential underground reservoirs. This would allow oil and gas companies to more accurately predict the suitability of the hydrocarbons at potential drilling sites.

## Introduction

In the crude oil and gas industry, an accurate estimation of the pre-exploitation distribution of hydrocarbon in underground layers and sediments is one of the necessary prerequisites for a successful field plan development. This is not an easy task as petroleum fluids are very complex mixtures composed of a large number of species that are contained within a porous media under high pressure. The distribution of species in the reservoir is known to be influenced by many phenomena^1^; traditionally, gravitational segregation is assumed to be the most important one, at least in a closed, convection-free reservoir.^2^ More recently some authors highlighted thermodiffusion as another phenomenon that could be of major influence in determining the initial (pre-exploitation) distribution of species.^1, 3, 4^

Thermodiffusion, sometimes called thermophoresis or Soret effect in dense phase, is a phenomenon which was discovered more than a century ago^5, 6^ and is observed in gaseous, liquid and even solid mixtures when subjected to a temperature gradient. In a convection-free environment, the presence of a temperature gradient leads to a composition gradient in a mixture, as species preferentially migrate towards either cold or hot sections. This separation effect occurs also when fluids are confined in a porous medium, and it has been shown that the porous medium has usually a negligible effect on the magnitude of thermodiffusion, so that measurements of thermodiffusion in unconfined fluids can be used for most reservoir applications.^7–9^

Thermodiffusion is present in most reservoirs because the fluids in place are subject, over geological times, to a vertical geothermal gradient^1^ of about 0.03 K/m. Furthermore, the fluids are confined in low permeability porous medium, which usually limits the onset of convection. In some specific gas or oil reservoirs, there is some evidence that thermodiffusion can be as important as gravitational segregation^3, 10–12^ and therefore it should not be neglected when modelling the initial distribution of species within reservoir fluids.

In the last 20 years, remarkable progress has been achieved in accurately measuring thermodiffusion coefficients in dense fluids.^13, 14^ However, experimental results are mostly limited to binary liquid mixtures at atmospheric pressure, notwithstanding some recent collaborative efforts that were devoted to measurements in ternary mixtures^14, 15^ and under high pressures.^16–19^ From the theoretical point of view, some progress has been achieved both in the field of thermodiffusion simulation^20^ and modelling.^14, 21^ Nevertheless, a number of open questions still exist regarding the limitations of the current models when applied to describe thermodiffusion in multicomponent petroleum fluids at typical reservoir conditions.

There is thus a clear need for reliable data on multicomponent mixtures under reservoir conditions, in order to provide benchmark reference data that can be used to validate and further develop models and simulation tools.^1^ Such experiments are difficult to perform not only because of the complexity of characterising the thermodiffusion in multicomponent mixtures, but also because under the normal laboratory conditions the magnitude of the effect is small. As stressed more than 20 years ago,^22^ performing measurements under microgravity conditions is one possible solution to generate data on thermodiffusion in multicomponent mixtures.^11, 23–25^

The need for accurate reference data was one of the main drivers behind this project 'Soret Coefficient measurements of Crude Oil' (SCCO) which, using a microgravity set up implemented in the SJ-10 satellite,^26^ aimed to measure the thermodiffusion coefficients at high pressures of six multicomponent fluid mixtures, of interest to reservoir applications. The results of this SCCO/SJ-10 microgravity experiment, flown in April 2016, are presented in this article and are compared with molecular dynamics simulations results. In addition, using thermodynamic modelling, the influence of thermodiffusion on the initial state of an idealised one-dimension gas condensate reservoir is evaluated.

## Results

### Experiments

The SCCO/SJ-10 microgravity experiment has been conducted on six different synthetic samples composed of linear alkanes. Binary, ternary and quaternary mixtures containing methane (C1), *n*-pentane (nC5), *n*-heptane (nC7) and *n*-decane (nC10) have been studied under high pressure in a monophasic state. Compositions and pressures used in the six different experimental cells are given in Table 1, with pressures corresponding to the in-flight average temperature of 50.8 °C at which experiments were performed. The SCCO/SJ-10 hardware and experimental design was similar to the one employed for the SCCO/Foton-M3 mission in 2007, and further details can be found elsewhere^11, 24^ as well as in the Materials and Methods section.

**Table 1:** Composition and pressure of the six SCCO cells embarked in SJ-10

Cell ID	Pressure (MPa)	Composition (% mole fraction is indicated between parenthesis)
A	31.1	nC5 (50), nC10 (50)
B	40.2	nC5 (50), nC10 (50)
C	31.0	nC5 (33.33), nC7 (33.33), nC10 (33.33)
D	40.1	nC5 (33.33), nC7 (33.33), nC10 (33.33)
E	35.0	C1 (96.5), nC5 (1.17), nC7 (1.17), nC10 (1.17)
F	40.0	C1 (96.5), nC5 (1.17), nC7 (1.17), nC10 (1.17)

From the gas chromatography analysis of cold and hot compartments of each SCCO cell after the flight, it appeared that some cells have suffered from leakages yielding incoherent results. This conclusion is confirmed independently by accurate weighing of filled cells before and after the flight. The leakage may have many root causes, but most of them can be attributed to either unpredictable thermal effects at those high pressures inside the cells (more than 30 MPa, see Table 1) or to unexpected landing shocks. In particular, cells A, B, D and F were found to be partially empty, to the extent that no sensible analysis could be performed and therefore they will not be discussed further. Results on the two exploitable cells (C and E), containing ternary and quaternary mixtures, are presented in Table 2.

**Table 2:** Measured GC composition (in % mole fraction) in the two exploitable SCCO-SJ-10 cells after the flight. Initial compositions (before the flight) are provided in Table 1

Cell ID	Cold compartment				Hot compartment			
Species	C1	nC5	nC7	nC10	C1	nC5	nC7	nC10
C	NA	30.63 ± 1.51	33.3 ± 0.33	36.07 ± 1.85	NA	35.85 ± 0.19	33.37 ± 0.25	30.78 ± 0.43
E	96.12 ± 0.07	1.17 ± 0.02	1.38 ± 0.01	1.35 ± 0.08	96.92 ± 0.56	1.16 ± 0.03	0.96 ± 0.04	0.98 ± 0.49

*NA*, not applicable (ternary mixture)

From the compositional variation in the two compartments, and assuming a linear response, it is possible to quantify thermodiffusion of the studied mixtures. To do so, in a multicomponent mixture, it is customary to use the so-called thermal diffusion ratio of each species, which is defined in this case in the linear response regime as:

1 $${k_{{T_i}}} = - T\frac{{\Delta {x_i}}}{{\Delta T}}$$

where *x*_*i*_ is the mole fraction of component *i*, *T* is the average temperature and Δ indicates difference between the two cell compartments. Such a computation requires knowledge of the average temperature difference between the two compartments of each cell for which, following the work of Van Vaerenbergh et al.,^24^ we adopted an estimation of Δ*T* = 12.45 ± 0.20 °C. SJ-10 results, presented in terms of the thermal diffusion ratio associated to each species, are provided in Table 3.

**Table 3:** Measured and simulated thermal diffusion ratio of the various species in the two exploitable SCCO-SJ-10 cells. Values in parenthesis correspond to the results of the AUA molecular model

Cell ID	Experimental ${{\boldsymbol{k}}_{{{\boldsymbol{T}}_{\boldsymbol{i}}}}}$				Simulated ${{\boldsymbol{k}}_{{{\boldsymbol{T}}_{\boldsymbol{i}}}}}$			
Species	C1	nC5	nC7	nC10	C1	nC5	nC7	nC10
C	NA	−1.36 ± 0.47	−0.02 ± 0.14	1.38 ± 0.62	NA	−0.09 ± 0.03 (−0.19 ± 0.07)	−0.09 ± 0.03 (0.05 ± 0.08)	0.18 ± 0.04 (0.14 ± 0.08)
E	−0.21 ± 0.17	0 ± 0.01	0.11 ± 0.09	0.1 ± 0.08	−0.16 ± 0.04	0.04 ± 0.01	0.05 ± 0.01	0.07 ± 0.02

*NA* not applicable (ternary mixture)

### Molecular dynamics simulations

To complement the microgravity experimental measurements, non-equilibrium molecular dynamics simulations of the thermal diffusion ratios of the fluid contained in cells C and E have been performed. Simulations were carried out using a recently developed Mie Chain Coarse-Grained (MCCG) molecular model.^27^ As thermodiffusion is known to be very sensitive to the force field, additional simulations using an Anisotropic United Atom molecular representation^28^ have been performed on the liquid (nC5–nC7–nC10) mixture. All results are given in Table 3.

### Thermodynamic modelling

**Fig. 1:**
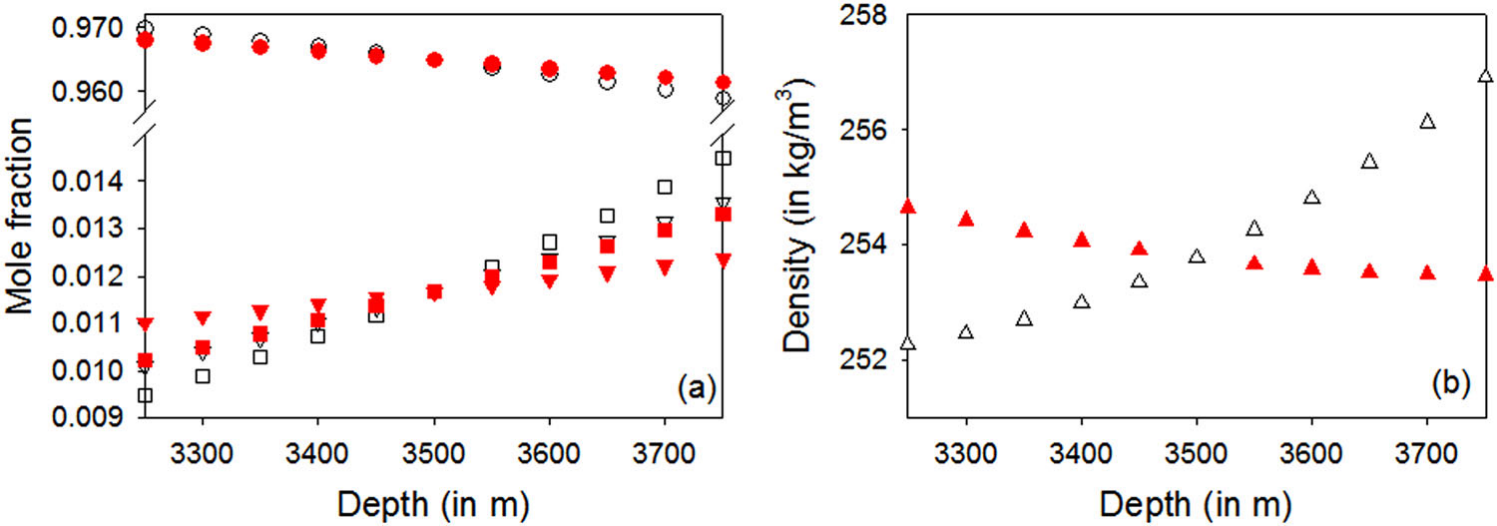
Simulated species distribution **a** and density **b** of a condensate gas (cell E) as a function of depth. *Open black symbol*: only gravitational segregation. *Solid red symbols*: thermodiffusion included in the model. Left panel **a**: C1 (*circles*), nC7 (*triangles*) and nC10 (*squares*)

As an illustration of the influence of thermodiffusion on the initial vertical distribution of species in a reservoir, simulations of the gas condensate (cell E composed of C_1_, nC_5_, nC_7_ and nC_10_, see Table 1) have been performed using a thermodynamic reservoir model combined with a cubic equation of state.^29^ More precisely, we have simulated a hypothetic fluid column located between 3250 and 3750 m of depth, and subjected to a geothermal gradient of 0.03 K/m. The reference point, at 3500 m depth, has been taken at the average thermodynamic conditions of cell E (i.e., *T* = 50.8°C and *P* = 35 MPa). Two different cases have been modelled. The first one in which only gravitational segregation has been taken into account and the second one in which both gravitational segregation and thermodiffusion (thermogravitation) have been included. For the second case, the experimental thermal diffusion ratios of Table 3 were used. Results on compositional and density variations with depth are shown in Fig 1.

## Discussion

Regarding SCCO-SJ-10 experiment, as shown in Table 2, the composition of the two compartments (cold and hot) of each exploitable cell is noticeably different as expected. Furthermore, results clearly show that, in both liquid (cell C) and gas condensate (cell E) mixtures, the lightest species had a tendency to migrate (relatively to the heaviest) to the hot compartment, while the heaviest migrated towards the cold end. Such behaviour is consistent with what has been known qualitatively for a long time^30^ and also with more recent experimental results on binary hydrocarbon mixtures.^13, 14^ In addition, it appears that the intermediate species, nC7 in the ternary mixture and nC5 in the quaternary mixture, are uniformly distributed showing only weakly migrating patterns. Such trend is consistent with what is known about the mass effect in non-binary mixtures.^31, 32^ Qualitatively, the experimentally observed behaviour is also consistent with the molecular simulations performed for this work, see Table 3, and with previous molecular dynamics results on multicomponent hydrocarbon mixtures^11, 12, 33, 34^ too.

From a quantitative point of view, the experimental thermal diffusion ratios for the quaternary mixture (gas condensate) are of the same order of magnitude as those obtained experimentally in binary mixtures of similar compounds.^13, 14, 35, 36^ The experimental results on the quaternary mixture are consistent with molecular dynamics predictions; see Table 3, which reinforces their overall reliability. One can nevertheless notice non-negligible differences between experimental and simulated data, and between the simulation results based on different force fields. This is not unexpected given the known high sensitivity of thermodiffusion to the force fields and the difficulties associated with the high-pressure microgravity experiments.

The experimental results on the ternary liquid mixture, see Table 3, are unexpected. Specifically, the experimental thermal diffusion ratios for nC5 and nC10 in this system are one order of magnitude larger than those obtained in the molecular dynamics simulations (irrespective of the force field) or those obtained for the same species in the gas condensate mixture. Since molecular simulations are able to estimate reasonably well thermal diffusion ratios in binary liquid mixtures composed of nC5 and nC10,^27, 28^ we can only conclude that the experimental results for the ternary liquid mixture have higher uncertainty than anticipated and that the cell C has likely suffered from unknown problems during the flight and/or analysis.

Regarding a possible influence of pressure on thermodiffusion, molecular simulations, using the coarse-grained model, on cell D (liquid at 40.1 MPa) and cell F (gas condensate at 40 MPa) have yielded results within the errors bars of cell C (liquid at 31 MPa) and E (35 MPa), respectively. This indicates a weak influence of pressure on thermal diffusion ratios in such systems consistently with what is known from experimental results in binary liquid mixtures.^19^

The introduction of the experimental thermal diffusion ratios of the quaternary mixture in the modelling of the initial vertical distribution of species of a synthetic reservoir reveals interesting features. As expected, gravitational segregation alone leads to an enrichment of the lightest compound at the top of the reservoir, whereas heavier compounds are enriched at the bottom, Fig. 1. More interesting is the case in which thermodiffusion is included (thermogravitational case). The left panel of Fig. 1 shows that, even if buoyancy is dominant, thermodiffusion tends to counteract the influence of gravitational segregation. Thus, omitting thermodiffusion in the modelling causes a noticeable overestimation of the concentration gradient, confirming earlier predictions for a different reservoir type (e.g., acid gas^12^). Even more striking are the density profiles on the right panel of Fig. 1. In the gravity-only case, density increases with depth, as expected. However, when thermodiffusion is included, the density gradient is reversed, which could result in an unstable fluid column. One concludes that, depending on the permeability of the reservoir and on a possible tilt of the thermal gradient relatively to the gravity field,^1^ convection may appear in such reservoir, leading to an even more homogenised vertical fluid composition. More generically, these results confirm that, in oil and gas exploitation, thermodiffusion data for multicomponent mixtures, such as those obtained during the SJ-SCCO experiment, are crucial for a correct evaluation of the initial state of a reservoir.

Further dedicated on ground experiments are in progress in order to complement the experimental results obtained in the microgravity environment of a space flight. Partial results have already been published^18, 19^ including experiments on non-equilibrium fluctuations in binary^37, 38^ and ternary mixtures.^39, 40^

## Materials and methods

### Experiments

**Fig. 2:**
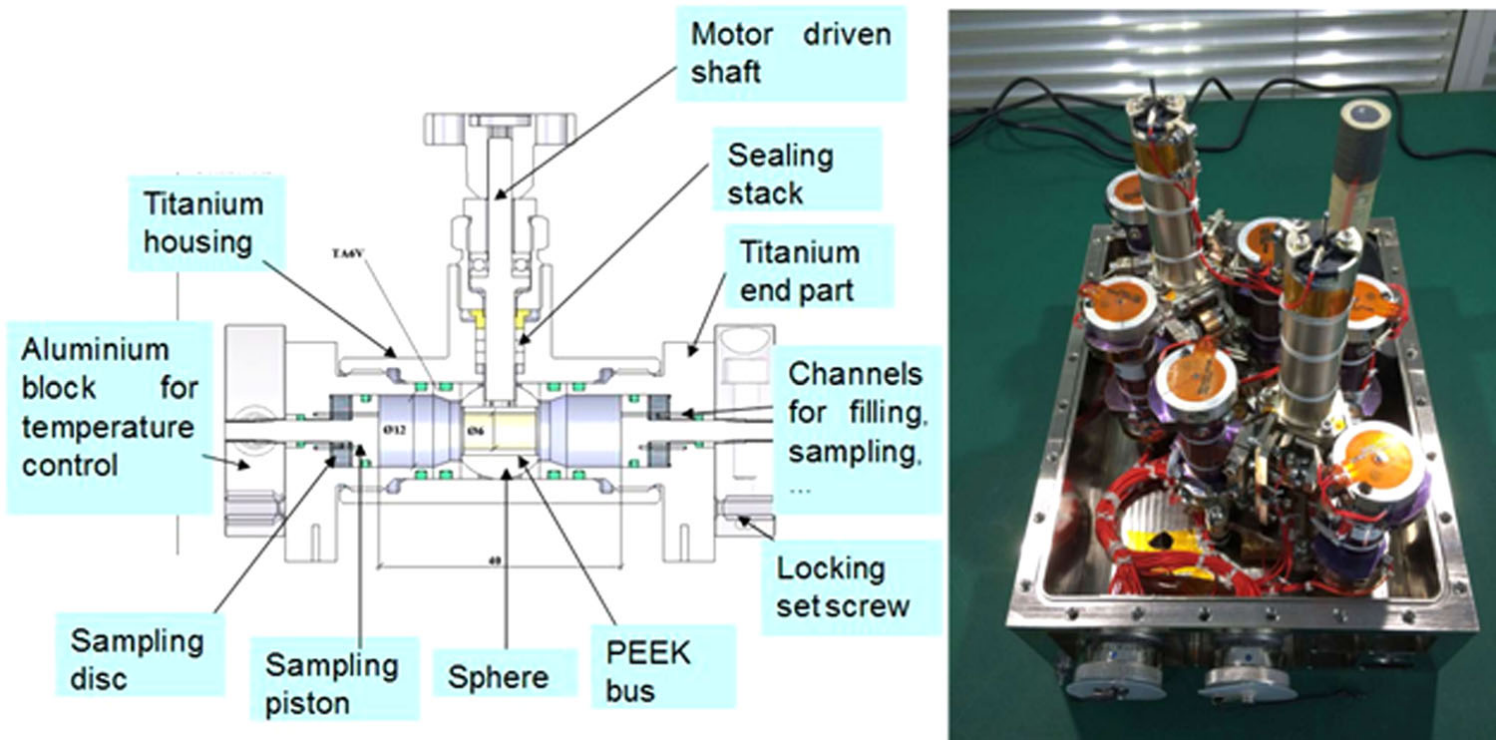
Cross-section view of the high pressure cell design (left figure) and photo of the C-box containing the six SCCO cells (right figure). The left figure has been created by Core Laboratories: Sanchez Technologies and the right picture has been taken by A.V.

The SCCO/SJ-10 mission was made possible by a partnership between the China National Space Administration (CNSA) and the European Space Agency (ESA). The experiment set up flown in the SJ-10 satellite consists of six small and sturdy titanium cells, containing 1.2 ml of fluid, divided into two halves that are linked by an initially open valve, Fig. 2. The cells were attached to two supporting triads and placed into a hermetically sealed aluminium crate, named C-box, Fig. 2. The cell and experiment designs, developed by Core Laboratories: Sanchez Technologies (Paris, France), are similar to the ones used during the Foton-M3 mission in 2007, and further details can be found elsewhere.^11, 24^

During the orbital flight, the two flat surfaces of every cylindrical cell were maintained at two different temperatures, so as to induce thermodiffusion inside the fluid contained therein. At the end of the flight, i.e., after 270 h of operational time, the central valves in all cells were closed shut separating each fluid sample into two fractions (a 'hot' and a 'cold' part).

A difference with the previous Foton-M3 mission is that the SJ-10 satellite was not pressurised. As a consequence, the C-box enclosing the six SCCO cells (which were specifically designed for Foton) had to be filled with nitrogen slightly above ambient pressure. Pressure inside the C-box was monitored by a manometer. The C-box is firmly screwed to the satellite external shield that is in contact with space, which provides a heat sink for temperature control since it is shadow oriented throughout the mission.

### Fluid preparation and cell filling process

**Fig. 3:**
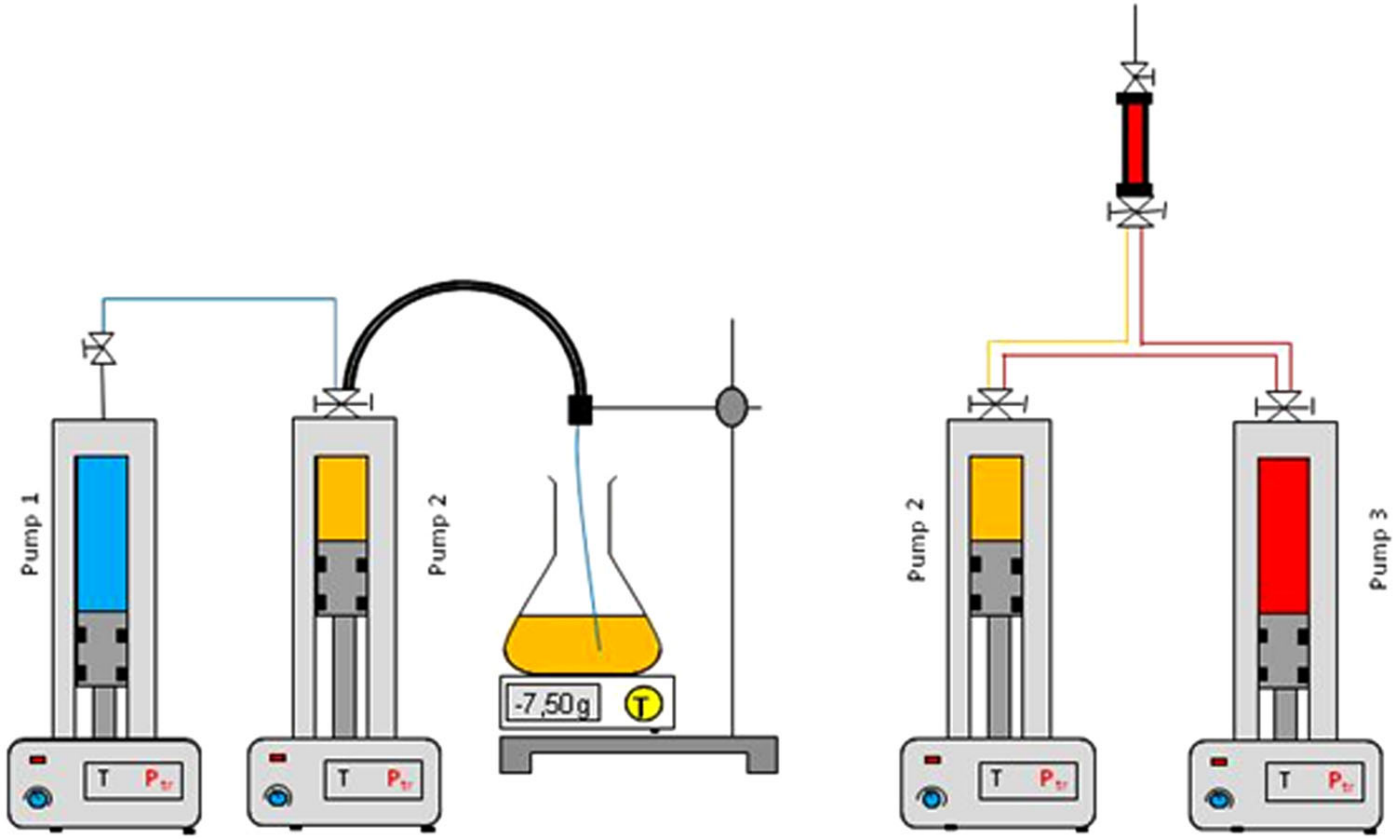
Sketches of the protocol for the liquid mixture preparation (left figure) and gas–liquid mixtures (right figure). This figure has been created by H.B.

Three STIGMA automatic pumps from Core Laboratories: Sanchez Technologies were used for sample preparation and cell filling procedures. Each pump controls the injection pressure, the injected volume and temperature. Injections have been performed at 20 °C under pressure chosen to reach the target values of Table 1 at the average operational temperature of 50.8 °C. The simple and sturdy design of SCCO did not allow for pressure sensors inside the cells, so that an equation of state needs to be used to estimate these filling pressures. Two different filling protocols, as described below and in Fig. 3, have been used depending on the mixture type.

The four liquid mixtures (A–D) were prepared in the following manner. First, nC5 was introduced into pump 1. Then, pre-evacuated pump 2 was filled with nC10 or with nC7-nC10 premix by aspiration and weight control. By measuring the volume, the desired amount of nC5 was introduced into pump 2 from pump 1. The mixtures were homogenised by integrated mechanical stirring and then transferred into the SCCO cells. The cell volume was swept at least three times by the sample. The cell pressure was adjusted and controlled by pump 2.

Although at the target temperature of 50.8 °C the quaternary mixtures of cells E and F are in homogeneous phase, at the filling temperature of 20 °C they are phase-separated. Hence, a specific protocol was designed for their preparation, and the two gas condensate mixtures (E and F) were prepared in the following manner: Pump 2 was initially filled with nC5-nC7-nC10 equimolar ternary mixture using the procedure described above. Next, methane was introduced into pump 3 at a pressure of 27.5 MPa. The SCCO cells were first filled with pure C1 at 20 MPa. Then, from pump 3 the desired quantity of methane was transferred into pump 2. The quaternary mixture so obtained was homogenised and pressurised to 27.5 MPa and then transferred into the cells. The cells volume was swept at least five times by the sample. The cells pressure was adjusted and controlled by pump 2.

### Conditions during the SJ-10 orbital flight

On 6 April 2016 at 01:38 local time, a Long March (Chang Zheng) 2D rocket lifted-off from China’s Jiuquan Satellite Launch Centre carrying the SJ-10 (Shi Jian 10) research spacecraft. The satellite’s scientific payload entailed of a variety of experiments including SCCO. SJ-10 landed in the Inner Mongolia region north of Beijing on 18 April at 15:04, a bit more than 12.5 days after its launch.

Once SJ-10 reached its stabilised orbit, on 7 April at exactly 14:00, the SCCO experiment was powered on, starting the slow but steady segregation of chemical species within its tiny load. Inside the C-box, a set of heaters and Peltier elements maintained stable temperatures at the two extremities of each cells, equal to 35.85 ± 0.05 °C and 65.88 ± 0.05 °C, respectively, i.e., an average thermal gradient of about 0.445 K/mm. After activation and the initial transitory phase, the target temperatures of the two triads were met in about 40 min. Gravity levels inside the satellite during SCCO operation on SJ-10 were about 10^−6^–10^−5^ *g* for quasi-steady state (<0.1 Hz), except for some short time orbit control during which the gravity level was about 10^−3^ *g*. The C-Box is air tight and its inner pressure was continuously monitored. After a short transient, the pressure stayed at a value of 0.11 ± 0.01 MPa.

On 17 April at 21:09, the temperature control at the flat surfaces of the six cylindrical cells was automatically switched-off, following the closure of the intercepting valves about 12 min earlier. The subsequent slow decrease in temperature at both ends of the HP cells had no effect on the mixture composition, as the fluids remained safely confined within the two separate halves of the cells.

### Cell analysis process

Immediately after satellite recovery, the C-box containing the SCCO samples was forwarded to the Petrochina laboratory at Beijing for analysis. The time that elapsed since satellite recovery and the beginning of the analysis procedure was of the order of 1 day.

Two Agilent Gas Chromatographs 6890 N have been used to analyse the six samples. The reported compositions in Table 2 correspond to an average between two different GC injections for each compartment and the associated error bars correspond to half the difference between the results of the two injections. The composition results were reproducible to within 2% of mole fraction of a given compound. It should be pointed out that the GC protocol has been tested on a cell that stayed on earth and contained the same quaternary mixture as cells E and F. For GC analysis, and depending on the phase of the mixture, two different protocols have been used.

For binary and ternary liquid mixtures (A–D), the first step was to store the cells at −15 °C in order to limit evaporation of the lightest alkane (nC5). Following this pre-conditioning, the two compartments of each cell have been emptied using dedicated syringes and the extracted fluids were then stored in Agilent vials with volume redactor. When the extracted volume was insufficient, the liquid samples were diluted in a solvent (carbon disulphide) which did not affect the analysis. Finally, the fluid samples have been injected in the gas chromatograph and analysed.

For quaternary gas condensate mixtures (E and F) the first step consisted of diluting the fluids contained in the two compartments of each cell, by using hydrogen, so as to avoid phase separation. The resulting diluted mixtures were initially stored in a dedicated reservoir with a volume of 1 l, and subsequently transferred to the GC for analysis. The reservoirs have been heated to the same temperature as the GC line, in order to avoid cold points.

### Molecular simulations

#### Non-equilibrium molecular dynamics

Molecular simulations have been achieved using Boundary-Driven Non-Equilibrium Molecular Dynamics (BD-NEMD) schemes.^41, 42^ These methods are well suited to deal with thermal diffusion ratio in multicomponent mixtures.^32, 33^ In such approach, one mimics a real experiment by imposing a huge heat flux (but still in the linear response regime)^41–43^ to the simulation box containing the studied mixture. This produces a thermal gradient of about 1 K/Å.^42^ This induces, after a transient state, mole fraction gradients parallel to the temperature gradient because of thermodiffusion. The temperature gradient and species mole fraction gradient are then obtained from the slope of a linear regression of the simulation data. Hence, it is possible to directly estimate thermal diffusion ratios from Eq. (1). Two different force fields to represent the molecules have been used in the computations, as described below.

#### Coarse-grained simulations

The coarse-grained molecular model used in this work consists of representing the studied compounds by simple homo-nuclear chain made up of spheres freely and tangentially bonded.^44^ Two adjacent spheres in a chain are linked by a rigid bond and the interaction between two non-bonded spheres is described by the Mie n-6 potential. A cutoff radius equal to 3.5σ has been used and long-range corrections have been included.^45^ In such coarse-grained representation, methane is modelled by a monomer, *n*-pentane by a dimer, *n*-heptane by a trimer and *n*-decane by a tetramer. Force field parameters have been assigned using the procedure described in Hoang et al.^27^

Simulations have been performed using the BD-NEMD scheme proposed in ref. 42 and the Verlet velocity algorithm has been used to integrate the equations of motion. To constrain the bond length, we have used the classical RATTLE algorithm.^45^ Averaged temperature and pressure have been controlled using the Berendsen approach.^45^ Simulations have been performed on systems composed of 1000 to 3600 molecules. To compute thermal diffusion ratios, averages have been achieved using six independent runs of 2×10^7^ non-equilibrium time steps. Error bars have been estimated using the sub-block method.^45^

#### Anisotropic united atoms simulations

The model used in these simulations is the Anisotropic United Atoms model^46^ AUA4. This model has already been validated against experimental thermodiffusion data in binary mixtures.^28^ In the AUA4 model, CH_2_ and CH_3_ groups are represented by Lennard–Jones particles and the hydrogen atoms are implicitly taken into account by shifting the centre of force from the carbon atom towards hydrogen atoms by a distance *δ*. Alkane flexibility is reproduced using bending and torsional contributions while carbon–carbon bond lengths are kept constant using the RATTLE algorithm. Details of the model can be found in ref. 46.

Our in-house code NEWTON was used for all simulations. Equations of motion were integrated out using the velocity Verlet scheme with a time step of 1 fs. A spherical cutoff of 12 Å is used and long-range corrections are applied to energy and pressure, considering a uniform distribution for distances above the cutoff.^45^ Periodic boundary conditions are applied in all directions. The heat exchange algorithm^41^ was used to compute the thermal diffusion in the binary Lennard–Jones mixtures. Simulations on this mixture were conducted on 3000 molecules (22,000 particles) and local temperature and composition were accumulated for at least 30 ns at the stationary state to compute the thermal diffusion ratio. Error bars have been estimated using the sub-block method.^45^

### Thermodynamic modelling

Thermodynamic modelling of both gravitational segregation (with a geothermal gradient of 3 K/100 m) and thermogravitation (gravitational segregation + thermodiffusion) have been achieved using a procedure described in Galliero and Montel.^29^ The approach is based on a numerical procedure to solve an extended Gibbs equation for each species *i*:

2 $$\nabla {\mu _i} = - {M_i}{\boldsymbol{g}} - {Q_i}\frac{{\nabla T}}{T}$$

where *μ*_i_ is the chemical potential, *M*_i_ the molecular weight, *g* the gravitational acceleration and *Q*_i_ is the so-called heat of transport, which is related to the thermal diffusion ratio.^29^ For the present work, the values of *Q*_i_ were tuned simultaneously in order to match exactly the measured compositions from Table 3. A constraint was added to minimise the pressure difference between hot and cold side. Because all compositions are fixed, this pressure difference is not exactly zero at the minimum of the objective function. This means that the measured compositions are not fully thermodynamically consistent.

The optimised values of *Q*_*i*_ thus obtained were used in the prediction of the reservoir fluid column gradient. Once combined with the mechanical equilibrium of the fluid column, Eq. (2) allows to compute the composition at any given point of a closed reservoir, as described and validated in Galliero and Montel.^29^

To model the chemical potential of each species, Peng–Robinson equation of state (PR-EOS) with volume shift has been applied.^47^ The volume shift of each component has been adjusted in order to get the exact molar volume at reservoir conditions using NIST reference Database.^48^ The binary cross interactions parameters, relevant for PR-EOS, have been obtained using the Jaubert and Mutelet method.^49^

### Data availability

The data that support the findings of this study are available from the authors on request.

## References

[1] Montel F, Bickert J, Lagisquet A, Galliero G (2007). Initial state of petroleum reservoirs: a comprehensive approach. J. Pet. Sci. Eng..

[2] Sage BH, Lacey WN (1939). Gravitational concentration gradients in static columns of hydrocarbons fluids. Petr. Trans. AIME.

[3] Holt, T., Lindeberg E. & Ratkje, K. S. The effect of gravity and temperature gradients on methane distribution in oil reservoirs. SPE Paper 11761 (1983).

[4] Whitson, C. H. & Belery, P. Compositional gradients in petroleum reservoirs. SPE Paper 28000 (1994).

[5] Ludwig C (1856). Diffusion zwischenungleicherwärmtenOrtengleichzusammengesetzterLösungen. Sitzber. Akad. Wiss. Wien. Math. Naturw. Kl..

[6] Soret C (1879). Arch. Sci. Phys. Nat..

[7] Shapiro A, Stenby EH (2000). Factorization of transport coefficients in macroporous media. Transp. Porous Media.

[8] Platten JK, Costeseque P (2004). The Soret coefficient in porous media. J. Porous Media.

[9] Hannaoui R, Galliero G, Hoang H, Boned C (2013). Influence of confinement on thermodiffusion. J. Chem. Phys..

[10] Ghorayeb K, Firoozabadi A, Anraku T (2003). Interpretation of the unusual fluid distribution in the Yufutsu gas-condensate field. SPE J..

[11] Touzet M (2011). Thermodiffusion: from microgravity experiments to the initial state of petroleum reservoirs. Comptes Rendus Mecanique.

[12] Galliero G (2016). Impact of thermodiffusion on the initial vertical distribution of species in hydrocarbon reservoirs. Microgravity Sci. Technol..

[13] Wiegand S (2004). Thermal diffusion in liquid mixtures and polymer solutions. J. Phys. Condens. Matter.

[14] Köhler W, Morozov KI (2016). The soret effect in liquid mixtures – a review. J. Non-Equilib.Thermodyn..

[15] Bou-Ali MM (2015). Benchmark values for the Soret, thermodiffusion and molecular diffusion coefficients of the ternary mixture tetralin + isobutylbenzene + n-dodecane with 0.8-0.1-0.1 mass fraction. Eur. Phys. J. E Soft Matter.

[16] Urteaga P (2012). Measurement of thermodiffusion coefficient of hydrocarbon binary mixtures under pressure with the thermogravitational technique. Rev. Sci. Instrum..

[17] Giraudet C, Bataller H, Croccolo F (2014). High-pressure mass transport properties measured by dynamic near-field scattering of non-equilibrium fluctuations. Eur. Phys. J. E Soft Matter.

[18] Lizarraga I, Giraudet C, Croccolo F, Bou-Ali MM, Bataller H (2016). Mass diffusion and thermal diffusivity of the decane-pentane mixture under high pressure as a ground-based study for SCCO project. Microgravity Sci. Technol..

[19] Lizarraga I, Croccolo F, Bataller H, Bou-Ali MM (2017). Soret coefficient of the n-dodecane –n-hexane binary mixture under high pressure. Eur. Phys. J. E Soft Matter.

[20] Artola PA, Rousseau B (2013). Thermal diffusion in simple liquid mixtures: what have we learnt from molecular dynamics simulations?. Mol. Phys..

[21] Assael, M. J., Goodwin, A. R. H., Vesovic, V. & Wakeham, W. A. *Experimental Thermodynamics Volume IX: Advances in Transport Properties of Fluids*. (Royal Society of Chemistry, 2014).

[22] Legros JC, Van Vaerenbergh S, Decroly Y, Montel F (1994). Expériences en microgravité étudiant l’effet Soret: SCM, SCCO et MBIS. Entropy.

[23] Georis, P., Montel, F., Van Vaerenbergh, S., Decoly, Y. & Legros, J. C. *Proceedings of the European Petroleum Conference*, Society of Petroleum Engineers, SPE-50573-MS, vol. 1, 57–62 (1998).

[24] Van Vaerenbergh S, Srinivasan S, Saghir MZ (2009). Thermodiffusion in multicomponent hydrocarbon mixtures: experimental investigations and computational analysis. J. Chem. Phys..

[25] Khlybov OA, Ryzhkov II, Lyubimova TP (2015). Contribution to the benchmark for ternary mixtures: measurement of diffusion and Soret coefficients in 1,2,3,4-tetrahydronaphthalene, isobutylbenzene, and dodecane onboard the ISS. Eur. Phys. J. E Soft Matter.

[26] Hu WR (2014). Space program SJ10 of microgravity research. Microgravity Sci. Technol..

[27] Hoang H., Delage-Santacreu S. & Galliero G. Simultaneous description of equilibrium, interfacial and transport properties of fluids using a Mie Chain Coarse-Grained Force Field, *Ind. Eng. Chem. Res*. doi:10.1021/acs.iecr.7b01397

[28] Perronace A, Leppla C, Leroy F, Rousseau B, Wiegand S (2002). Soret and mass diffusion measurements and molecular dynamics simulations of n-pentane–n-decane mixtures. J. Chem. Phys..

[29] Galliero, G. & Montel, F. Understanding Compositional Grading in Petroleum Reservoirs thanks to Molecular Simulations, SPE Paper No. 12902 (2009).

[30] Kramers H, Broeder JJ (1948). Thermal diffusion as a method for the analysis of hydrocarbon oils. Anal. Chim. Acta.

[31] Galliero G, Montel F (2008). Nonisothermal gravitational segregation by molecular dynamics simulations. Phys. Rev. E.

[32] Artola PA, Rousseau B (2015). Isotopic effect in ternary mixtures: theoretical predictions and molecular simulations. J. Chem. Phys..

[33] Galliero G, Duguay B, Caltagirone JP, Montel F (2003). On thermal diffusion in binary and ternary mixtures by non-equilibrium molecular dynamics. Philos. Mag..

[34] Galliero G, Srinivasan S, Saghir MZ (2009). Estimation of thermodiffusion in ternary alkane mixtures using molecular dynamics simulations and an irreversible thermodynamic theory. High. Temp. High. Press..

[35] Alonso De Mezquia D, Bou-Ali MM, Madariaga JA, Santamaria C (2014). Mass effect on the Soret coefficient in n-alkane mixtures. J. Chem. Phys..

[36] Alonso de Mezquia D (2014). Thermodiffusion, Molecular Diffusion and Soret Coefficient of Binary and Ternary Mixtures on n-Hexane, n-Dodecane and Toluene. Eur. Phys. J..

[37] Ortiz de Zarate, J. M., and Sengers, J. V., *Hydrodynamic Fluctuations in Fluids and Fluid Mixtures* (Elsevier Science, 2006).

[38] Croccolo F, Ortiz de Zárate JM, Sengers JV (2016). Colloquium: non-local fluctuation phenomena in liquids. Eur. Phys. J. E Soft Matter.

[39] Bataller H, Giraudet C, Croccolo F, Ortiz de Zárate JM (2016). Analysis of non-equilibrium fluctuations in a ternary liquid mixture. Microgravity Sci. Technol..

[40] Martinez Pancorbo P, Ortiz de Zárate JM, Bataller H, Croccolo F (2017). Gravity effects on Soret induced nonequilibrium fluctuations in ternary mixtures. Eur. Phys. J. E Soft Matter.

[41] Hafskjold B, Ikeshoji T, Ratkje SK (1993). On the molecular mechanism of thermal diffusion in liquids. Mol. Phys..

[42] Reith D, Müller-Plathe F (2000). On the nature of thermal diffusion in binary Lennard-Jones Liquids. J. Chem. Phys..

[43] Kjelstrup S, Bedeaux D, Inzoli I, Simon JM (2008). Criteria for validity of thermodynamic equations for non-equilibrium molecular dynamics simulations. Energy.

[44] Mejia A, Herdes C, Müller EA (2014). Force fields for coarse-grained molecular simulations from a corresponding states correlation. Ind. Eng. Chem. Res..

[45] Allen M. P. & Tildesely D. J. *Computer Simulation of Liquids*, (Clarendon Press, 1987).

[46] Ungerer, P. et al Optimization of the anisotropic united atoms intermolecular potential for n-alkanes. *J. Chem. Phys.* **112**, 5499–5510 (2000). 10.1063/1.2219114 16942166

[47] Firoozabadi, A. *Thermodynamic of Hydrocarbon Reservoirs*. (McGraw-Hill, 1999).

[48] Jaubert JN, Mutelet F (2004). VLE prediction with the Peng-Robinson equation of state and temperature dependent kij calculated through a group contribution method. Fluid Phase Equilib..

[49] Jaubert, J. N. & Mutelet, F. VLE prediction with the Peng-Robinson equation of state and temperature dependent kij calculated through a group contribution method. *Fluid Phase Equilib.* **224**, 285 (2004).

